# Nucleolaron1511 and Nucleolaron1510 Facilitate Effective Intracellular Delivery of Azami Green Protein in HeLa Cells

**DOI:** 10.3390/ijms27156726

**Published:** 2026-07-28

**Authors:** Khai Nguyen, Ryo Yonezawa, Nahoko Bailey-Kobayashi, Tetsuhiko Yoshida, Shuichi Asakawa

**Affiliations:** 1 Laboratory of Aquatic Molecular Biology and Biotechnology, Department of Aquatic Bioscience, Graduate School of Agricultural and Life Sciences, The University of Tokyo, Bunkyo, Tokyo 113-8657, Japan; khainguyen98@g.ecc.u-tokyo.ac.jp (K.N.); ryonezawa@g.ecc.u-tokyo.ac.jp (R.Y.); 2 International Program in Agricultural Development Studies, Graduate School of Agricultural and Life Sciences, The University of Tokyo, Bunkyo, Tokyo 113-8657, Japan; 3 Signal Peptidome Research Laboratory, Department of Aquatic Bioscience, Graduate School of Agricultural and Life Sciences, The University of Tokyo, Bunkyo, Tokyo 113-8657, Japan; 4 Institute for Advanced Sciences, Toagosei Co., Ltd., Tsukuba, Ibaraki 300-2611, Japan; nahoko_kobayashi@mail.toagosei.co.jp (N.B.-K.); tetsuhiko_yoshida@mail.toagosei.co.jp (T.Y.)

**Keywords:** Nucleolaron, cell-penetrating peptide, Azami Green, TAT, fluorescent protein, recombinant protein, in vitro translation, intracellular delivery

## Abstract

The intracellular delivery of large macromolecules remains a significant challenge in both therapeutic and research contexts. This study investigates two novel chimeric cell-penetrating peptides, Nucleolaron1511 (Nuc1511) and Nucleolaron1510 (Nuc1510), with the aim of evaluating their capacities to deliver the fluorescent protein Azami Green (AG) into HeLa cells via genetic fusion. Cellular uptake of TAMRA-labeled Nuc1510 was assessed by fluorescence microscopy at multiple time points. Delivery efficiency of AG–Nuc1511 and AG–Nuc1510 fusion proteins was evaluated by fluorescence microscopy and flow cytometry, and cytotoxicity was assessed across a range of concentrations using the MTT assay. TAMRA–Nuc1510 successfully penetrated HeLa cells within one hour of treatment, with signal detected in both cytoplasmic and nuclear compartments. Flow cytometry quantification revealed that fusion with Nuc1511 increased intracellular AG fluorescence by over 80-fold relative to unconjugated AG, while Nuc1510 fusion yielded approximately a 9-fold increase. Neither peptide exhibited significant cytotoxicity at concentrations used in the delivery assays. These findings establish Nuc1511 and Nuc1510 as effective CPPs capable of facilitating intracellular protein delivery via genetic fusion, with favorable safety profiles, representing promising candidates for protein-based intracellular delivery in research and therapeutic applications.

## 1. Introduction

The plasma membrane serves as a dynamic and selective barrier governing molecular exchange between the cell and its environment, thereby maintaining cellular homeostasis. While small, nonpolar molecules diffuse freely across the lipid bilayer, the transport of large or hydrophilic macromolecules is tightly regulated. Consequently, the intracellular delivery of exogenous materials—including proteins, nucleic acids, and therapeutic compounds—remains a fundamental challenge in both basic research and clinical applications.

Cell-penetrating peptides (CPPs) have emerged as a promising strategy to address this challenge. The field was pioneered in the late 1980s with the discovery that the HIV-1 trans-activating protein (TAT) could enter cells and localize to the nucleus [1], followed shortly by the demonstration that the homeodomain of the Drosophila melanogaster protein Antennapedia possesses similar membrane-translocating properties [2]. Since these initial discoveries, an expanding repertoire of naturally occurring CPPs has been identified, and numerous artificial CPPs have been rationally designed and characterized, with poly-arginine and poly-lysine sequences among the most extensively studied [3,4].

CPPs possess the remarkable capacity to traverse cellular membranes and facilitate the intracellular delivery of diverse cargo molecules [5,6]. Key advantages include relatively low cytotoxicity, broad applicability across cell types, and dose-dependent cellular uptake [7]. CPPs have been shown to deliver a wide range of biologically relevant cargoes, including proteins [8,9,10,11,12], peptides [13,14], DNA [15,16], siRNA [17,18,19,20], and small-molecule drugs [21]. The versatility and relative simplicity of these approaches make CPPs a highly attractive tool in cellular and molecular biology. Despite extensive investigation, the mechanisms by which CPPs traverse the plasma membrane remain incompletely understood [22]. It is widely accepted that CPPs can enter cells via multiple pathways, including direct membrane penetration and endocytic uptake mediated by clathrin, caveolae, and other molecular machinery [23,24,25,26].

A previous study demonstrated that the nucleolar localization signal (NoLS) of LIM kinase 2 (LIMK2) functions as a CPP capable of delivering short peptides and green fluorescent protein into multiple cell types both in vitro and in vivo [27]. Building on these findings, we developed two novel chimeric CPPs, designated Nucleolaron1511 (Nuc1511) and Nucleolaron1510 (Nuc1510), with amino acid sequences TLKERCLQVVRSLVKKKRTLRKNDRKKR and KVLSRVVQLCREKLTRRRRSNRR, respectively (Figure 1A). The numerical designations of Nuc1511 and Nuc1510 reflect the order in which the peptides were synthesized. Both peptides incorporate the BC-box motif of the VHL protein, the inclusion of which was motivated by our prior observation that this motif promotes neuronal differentiation from neural stem cells [28]. Because the BC-box motif alone does not enter cells, we evaluated several chimeric constructs combining it with known CPP sequences, including TAT and the LIMK2 NoLS. Among these, the BC-box–LIMK2 NoLS combination, designated Nuc1511, exhibited particularly strong neuronal differentiation-inducing activity in neural stem cells and appeared to outperform LIMK2 NoLS alone in cellular uptake assays [29]. As neuronal differentiation-inducing activity in this context likely requires cytosolic delivery of the BC-box motif, we interpreted superior differentiation-inducing activity as indicative of enhanced cell permeability, and therefore selected Nuc1511 for further characterization. Nuc1510 was developed as a structural variant in which the BC-box motif is presented in reverse orientation and the LIMK2 NoLS is replaced by a distinct proprietary CPP sequence. These modifications are hypothesized to alter the charge density and amphipathic character of the peptide relative to Nuc1511, potentially influencing membrane interaction and uptake efficiency.

The present study extends our previous characterization of Nuc1511 by demonstrating its ability to deliver a larger protein cargo—the fluorescent protein monomeric Azami Green (AG)—into a model cancer cell line via genetic fusion, and provides a direct comparison with the widely used TAT peptide. In addition, cold-temperature inhibition experiments provide insight into the uptake mechanism of Nuc1511, and cytotoxicity profiling represents a further characterization not previously reported. Nuc1510 is described and characterized here for the first time, including assessment of its intrinsic cell-penetrating ability and protein delivery efficiency relative to both TAT and Nuc1511.

The results demonstrate that TAMRA–Nuc1510 effectively penetrates HeLa cells within one hour of treatment. Fusion with Nuc1511 increased intracellular AG fluorescence approximately 85-fold relative to unconjugated AG, while Nuc1510 fusion yielded approximately a 9-fold increase. Cytotoxicity was observed only at elevated concentrations for both peptides, with no significant effect on cell viability at concentrations up to 5 µM. Taken together, these findings establish Nuc1511 and Nuc1510 as effective CPPs for intracellular protein delivery via genetic fusion, representing promising additions to the intracellular delivery toolkit for research and therapeutic applications.

## 2. Results

### 2.1. Cellular Uptake of TAMRA-Labeled Nuc1510

To examine the intrinsic cell-penetrating ability of Nuc1510, HeLa cells were treated with TAMRA-labeled Nuc1510 prepared in medium supplemented with 10% FBS and analyzed by fluorescence microscopy at 1 h, 4 h, and overnight incubation time points.

At 1 h, TAMRA fluorescence was detected in nearly all cells and was primarily distributed diffusely throughout the cytosol, consistent with direct translocation across the plasma membrane (Figure 2A). At 4 h and overnight, discrete puncta became more prominent, suggestive of an increasing contribution from endocytic uptake at later time points. This shift was accompanied by an apparent weakening of the diffuse cytosolic signal, and after overnight incubation, the majority of signal was concentrated in small puncta outside of the nuclear region.

Single-section imaging at 1 h revealed discrete red puncta within the nucleus that did not colocalize with the DAPI signal, suggesting nucleolar localization of the peptide (Figure 2B). Consistent with this, the fluorescence intensity profile showed peaks in TAMRA signal coinciding with a sharp decline in DAPI signal, indicating accumulation within a DAPI-poor nuclear region. At 4 h and overnight, distinct nuclear puncta were no longer clearly observed, and the intensity profiles no longer exhibited the same elevated TAMRA signal at positions of sharp DAPI decline (Figure S1B).

Free TAMRA negative control showed no detectable intracellular fluorescence under any incubation condition (Figure S1A), confirming that the observed signal is attributable to Nuc1510, rather than to the fluorophore alone.

Together, these results demonstrate that Nuc1510 rapidly traverses the plasma membrane and accumulates in both cytoplasmic and nuclear compartments, with evidence of nucleolar localization.

### 2.2. Intracellular Delivery of AG-CPP Fusion Proteins in HeLa Cells

To evaluate the capacity of Nuc1510 and Nuc1511 to deliver a larger, biologically active cargo into cells and to compare their delivery efficiency, both peptides were genetically fused to the fluorescent protein monomeric Azami Green (AG). AG–TAT served as a positive control, and AG absent of a CPP served as a negative control. DNA cassettes were constructed by two-step PCR (Figure S2) and expressed using an E. coli cell-free in vitro translation system, followed by anti-His-tag magnetic bead affinity purification and buffer exchange into D-PBS; successful amplification, expression, and purification of all four constructs (AG, AG–Nuc1511, AG–Nuc1510, and AG–TAT) are shown in Figure S3.

HeLa cells were incubated overnight with each construct at 1, 2, or 3 µM prepared in complete culture medium supplemented with 10% FBS. Following incubation, cells were analyzed by fluorescence microscopy to evaluate uptake and by flow cytometry for quantitative assessment of intracellular AG signal.

Fluorescence microscopy revealed intracellular localization of AG signal for all three CPP fusion proteins (Figure 3A), with green fluorescent signal observed within the cell boundaries. At 3 µM, AG–Nuc1511 and AG–Nuc1510 appeared notably brighter than AG–TAT despite being imaged at lower exposure settings, suggesting higher intracellular accumulation. Intracellular delivery of AG fused with Nuc1511 and Nuc1510 was further supported by single-section imaging (Figure 3B), with additional single-section images of cells treated with these two constructs presented in Figure S4. HeLa cells treated with increasing concentrations of AG–CPP fusion proteins showed a corresponding increase in intracellular green fluorescence intensity (Figure S5), consistent with dose-dependent uptake. Between the two Nuc fusion proteins, AG–Nuc1511 displayed stronger signal intensity and labeling in a greater proportion of cells, indicating superior uptake efficiency relative to AG–Nuc1510.

The intracellular signal for both AG–Nuc1511 and AG–Nuc1510 appeared predominantly punctate, suggesting endocytic uptake as the primary entry mechanism. This interpretation is further supported by the near-complete abolition of delivery under 4 °C conditions (Figure S6), which arrests energy-dependent cellular processes. Notably, the puncta appeared markedly larger in AG–Nuc1511-treated cells than in AG–Nuc1510-treated cells, suggesting possible differences in intracellular trafficking or preferred endocytic mechanisms between the two fusion proteins, though further investigation would be needed to clarify this. In addition to discrete intracellular puncta, regions of more intense, irregularly shaped fluorescence were observed within the cell boundary (Figure S5). As this signal was absent in cells treated with unconjugated AG, we attribute these patches to membrane deformation occurring during the uptake of AG–CPP fusion proteins, although other underlying mechanisms cannot be excluded.

Notably, the single-section images and their corresponding line-scan fluorescence profiles showed that both the AG–Nuc1511 and AG–Nuc1510 signals were absent from the nuclear region of the cells (Figure 3B). This contrast with the nucleolar localization observed for TAMRA-labeled Nuc1510 (Section 2.1) suggests that fusion to different cargoes may alter the biophysical properties of the peptide in a manner that affects its uptake and subcellular routing. In contrast, AG–TAT showed nucleolar-targeting ability, as evidenced by discrete green puncta within the nucleus that did not overlap with DAPI signal (Figure S7).

For quantitative analysis, flow cytometry was used to measure intracellular AG fluorescence. Following overnight incubation, cells were washed twice with acidic glycine-NaCl solution (0.2 M glycine, 0.15 M NaCl, pH 3.0) to disrupt electrostatic interactions and remove surface-bound protein, then detached using trypsin. Flow cytometry quantification confirmed that fusion with each CPP significantly enhanced intracellular AG fluorescence relative to unconjugated AG (Figure 3C). AG–TAT yielded approximately 3.5-fold higher signal than the negative control, while AG–Nuc1510 and AG–Nuc1511 outperformed AG–TAT by approximately 2.5-fold and 24-fold, corresponding to roughly 9-fold and >80-fold increases over unconjugated AG, respectively. These findings are consistent with the fluorescence microscopy observations, collectively establishing the rank order of delivery efficiency as AG–Nuc1511 > AG–Nuc1510 > AG–TAT.

### 2.3. Cytotoxicity of AG–Nuc1511 and AG–Nuc1510 in HeLa Cells

To assess the cytotoxic potential of Nuc1511 and Nuc1510, HeLa cells were treated with AG–Nuc1511 or AG–Nuc1510 at concentrations of 1, 3, 5, and 10 µM for 24 h, and cell viability was quantified using the MTT assay (Figure 4). AG–Nuc1511 caused a gradual concentration-dependent decline in viability, reaching approximately 78% at 10 µM, which was statistically significant relative to the untreated control. AG–Nuc1510 did not significantly reduce viability at 1, 3, or 5 µM, but caused approximately 50% reduction at 10 µM, indicating cytotoxic effects at this elevated concentration. Taken together, both fusion proteins were well tolerated at concentrations up to 5 µM, with cytotoxicity becoming apparent only at 10 µM.

## 3. Discussion

The plasma membrane presents a fundamental barrier to the intracellular delivery of exogenous macromolecules, restricting the passage of large, hydrophilic, or charged species. CPPs have garnered considerable attention as a means to bypass this barrier, being capable of facilitating the intracellular delivery of diverse cargo types including proteins, nucleic acids, and small-molecule drugs [5,6]. In the present study, we extend our previous findings to design, produce, and characterize two chimeric CPPs, Nuc1511 and Nuc1510, evaluating their ability to deliver the fluorescent protein AG into HeLa cells via genetic fusion.

TAMRA-labeled Nuc1510 was employed to provide an initial characterization of the peptide’s intrinsic cell-penetrating ability, with the fluorophore serving as a low-molecular-weight tracer rather than a functional cargo. It should be noted that this experiment was not intended for direct comparison with previously published FITC-labeled Nuc1511 data, as TAMRA and FITC labeling differentially affect the physicochemical properties of peptides and would confound such comparisons. Fluorescence microscopy revealed a diffuse cytosolic signal at 1 h post-treatment, transitioning to a more punctate pattern at later time points. This temporal pattern suggests that Nuc1510 employs multiple entry mechanisms, with direct membrane translocation predominating at early time points and endocytic uptake contributing at later stages. The use of multiple entry pathways is a well-documented feature of CPPs, with the predominant mechanism heavily influenced by the target cell type, peptide concentration, and attached cargo [23,30,31,32,33].

Additionally, single-section imaging combined with fluorescence intensity line-scan analysis at 1 h revealed discrete puncta within the nucleus that did not overlap with DAPI signal, suggestive of nucleolar localization. This pattern was markedly less evident at later time points. The highly basic and polar nature of the Nuc1510 sequence resembles that of characterized nuclear and nucleolar localization signals [34,35], which may contribute to this transient nuclear accumulation, while the time-dependent decline of the nucleolar signal suggests gradual redistribution of the peptide from this compartment.

A comparable temporal pattern was reported by Tünnemann et al. [14] for TAT-mediated peptide delivery: TAT fused to a short peptide derived from hemagglutinin (HA2) showed a diffuse distribution and nucleolar localization within minutes of treatment in mouse myoblasts, but with prolonged observation the diffuse signal in the cytoplasm, nucleoplasm, and nucleoli became markedly weaker and vanished in most cells overnight, while peptide-containing cytoplasmic vesicles were unaffected and increased in number, eventually forming a vesicular ring around the nucleus. This suggests that small nucleolar-localizing peptides may undergo comparable time-dependent redistribution.

For direct comparison of delivery efficiency between Nuc1511 and Nuc1510, monomeric Azami Green was selected as a uniform cargo for both constructs. Both Nuc1511 and Nuc1510 showed a concentration-dependent uptake pattern, with signal intensity and the proportion of labeled cells increasing at higher concentrations. Dose-dependent uptake is a generally favorable characteristic of CPPs, allowing for more predictable control over delivery in various contexts [7].

Both AG–Nuc constructs displayed a punctate pattern within HeLa cells. AG–Nuc1511 appeared to be trapped within large, round vesicles, likely macropinosomes [36,37,38], whereas AG–Nuc1510 appeared trapped in smaller puncta resembling early endosomes. As uptake of both constructs was almost completely abolished under 4 °C conditions—which arrest all energy-dependent cellular processes—this observation further supports endocytosis as the predominant entry mechanism for both constructs. This is consistent with the broader CPP literature, in which endocytosis has been established as a predominant entry mechanism for many CPP–cargo fusion proteins, particularly those carrying large protein cargoes [33,39]. A detailed study employing selective endocytosis inhibitors to block caveolae-mediated, clathrin-mediated, and macropinocytic uptake should be conducted to further clarify the entry mechanisms of the two CPPs.

AG–Nuc1510 differs from TAMRA-labeled Nuc1510 in its intracellular localization. TAMRA–Nuc1510 showed nucleolar localization, whereas Nuc1510 fused to AG no longer demonstrated this subnuclear localization. Similarly, Nuc1511 fused to AG no longer demonstrated the capacity to bypass the nuclear membrane previously observed for FITC-labeled Nuc1511 [27]. Taken together, these observations highlight an effect of cargo identity on the intracellular trafficking of both Nuc1511 and Nuc1510. Cargo-dependent changes in CPP uptake mechanism and localization are a well-documented phenomenon [14,40,41]. Fusion to a cargo can substantially alter the physicochemical characteristics of a CPP; changes in polarity, charge density, hydrophobicity, or steric hindrance could all modify its interaction with negatively charged membrane components, thereby influencing uptake efficiency and intracellular localization. 

To quantify intracellular delivery, flow cytometry was performed following a stringent surface-depletion protocol. Cells were washed with an acidic glycine-NaCl solution to disrupt electrostatic interactions between surface-bound AG–CPP and the plasma membrane, then treated with trypsin, which both detaches cells from the culture plate and cleaves CPP sequences at arginine and lysine residues—amino acids abundant in all constructs tested—thereby eliminating residual surface-associated fluorescence [42,43,44]. This protocol ensures that the measured fluorescence predominantly reflects internalized protein. Flow cytometry quantification demonstrated that both Nuc1511 and Nuc1510 significantly outperformed TAT as a delivery vehicle, with AG–Nuc1511 and AG–Nuc1510 achieving approximately 24-fold and 2.5-fold greater intracellular fluorescence than AG–TAT, respectively, corresponding to >80-fold and ~9-fold increases over unconjugated AG. Patel et al. [45] compared the uptake efficiency of five common CPPs—TAT, Transportan, R8, Penetratin, and Xentry—fused to EGFP across four cell lines, finding that Transportan and R8 outperformed TAT by approximately 2.4-fold and 1.6-fold, respectively, while Penetratin performed comparably to TAT, and Xentry showed the lowest efficiency. In this context, based on the fold increase relative to TAT, the delivery efficiency of AG–Nuc1510 appears comparable to that of Transportan, while AG–Nuc1511 appears to surpass the five CPPs evaluated in that study. However, since the CPPs were not directly compared under identical experimental conditions, this comparison should be interpreted with caution, and future investigation of the Nuc CPPs should include these CPPs under the same experimental conditions to allow a more rigorous comparison.

Cell viability assessment by MTT assay confirmed that neither AG–Nuc1511 nor AG–Nuc1510 significantly affected HeLa cell viability at the concentrations used in the uptake assays, indicating a favorable safety profile for both constructs under these conditions. Cytotoxic effects were observed for both peptides at only 10 µM, a concentration exceeding that used in the delivery experiments. The difference in cytotoxicity between the two constructs at this elevated concentration may reflect their distinct charge distribution and amphipathic character, which could differentially affect interactions with cellular membranes and intracellular components. It should also be noted that the uptake, localization, and cytotoxicity of a CPP can vary with the attached cargo, so cytotoxicity is best evaluated in the context of the specific fusion protein under study.

Taken together, these findings establish Nuc1511 and Nuc1510 as effective CPPs for intracellular protein delivery via genetic fusion, with Nuc1511 demonstrating particularly high delivery efficiency. Several avenues remain for future investigation. Notably, although both CPPs demonstrated effective cellular uptake, the majority of the delivered cargo appeared to remain retained within endosomal compartments rather than being released into the cytosol. This limited endosomal escape represents a barrier to the practical application of these CPPs, as cargo retained within endosomes is unlikely to reach its intended subcellular target to exert biological function. Future studies should therefore investigate the endosomal escape efficiency of Nuc1511 and Nuc1510 in more detail and explore iterative design strategies aimed at improving this property. As noted above, the marked reduction in uptake observed under 4 °C conditions supports the use of selective endocytosis inhibitors—targeting, for example, clathrin-mediated endocytosis, caveolae-dependent pathways, or macropinocytosis—to help define the primary route of cellular entry for each construct. In addition, while this study focused on the delivery of a single protein cargo, the versatility of CPP-based delivery platforms suggests that Nuc1511 and Nuc1510 may also be capable of facilitating the intracellular delivery of other cargo types, including nucleic acids, drugs, and larger proteins, which warrants further investigation.

## 4. Materials and Methods

### 4.1. Cell Culture

HeLa cells (RIKEN, Ibaraki, Japan, RCB0191) were maintained in DMEM/F-12 (Thermo Fisher Scientific, Waltham, MA, USA) supplemented with 10% fetal bovine serum (FBS) and 100 µg/mL penicillin–streptomycin at 37 °C in a humidified atmosphere with 5% CO_2_. Cells were subcultured upon reaching 80–90% confluence.

### 4.2. Peptide Synthesis

TAMRA-labeled Nuc1510 (TAMRA–Nuc1510) was synthesized and purified to >90% purity by reverse-phase high-performance liquid chromatography (RP-HPLC) on a C18 column (Eurofins, Tokyo, Japan).

### 4.3. DNA Cassette Preparation for Cell-Free Expression

The coding sequence of Azami Green (AG) was obtained from the NCBI database, and the sequences of Nuc1511 or Nuc1510 were appended to its C-terminus via a linker sequence between the cargo and the CPP (amino acid sequence of the linker is GLQQGGTGGGGS). AG used as the negative control also has the linker sequence at its C terminus, but no CPP. The combined sequences were cloned into the pEX-A2J2 plasmid (Eurofins). DNA templates for cell-free in vitro translation of AG, AG–Nuc1511, and AG–Nuc1510 were generated by two sequential PCR reactions following the protocol of Yabuki et al. [46] with minor modifications (Figure S2A); the T7 promoter and terminator sequences used are shown in Figure S2B. The AG–TAT DNA template (with the T7 promoter and terminator fragments included) was commercially synthesized as a double-stranded DNA fragment (Eurofins) and amplified by PCR prior to use. Primer sequences are listed in Table S1. All PCR products were verified by 1.5% agarose gel electrophoresis, and DNA concentrations were quantified using the Qubit dsDNA Quantification Assay Kit (Thermo Fisher Scientific) and adjusted appropriately for downstream translation reactions. The complete amino acid sequences of AG, AG-Nuc1511, AG-Nuc1510, and AG-TAT are presented in the Supplementary Materials. 

### 4.4. Cell-Free Translation and Purification of AG–CPP Fusion Proteins

AG, AG–Nuc1511, AG–Nuc1510, and AG–TAT were synthesized using the Musaibo Kun N100 in vitro translation system (Taiyo Nippon Sanso, Tokyo, Japan). Translation reactions were carried out at 30 °C for 16 h in a thermoshaker (Farvogen Biotech Corporation, Ping Tung, Taiwan) at 300 rpm. Reactions were subsequently centrifuged at 15,000× *g* for 1 min at 4 °C, and the supernatant containing expressed protein was collected for purification.

Proteins were purified by Ni–IDA magnetic agarose bead affinity purification (MedChemExpress, Monmouth Junction, NJ, USA) according to the manufacturer’s instructions. Briefly, translation supernatant was incubated with three volumes of beads on a rotating mixer for 2 h at room temperature. Bound proteins were eluted in buffer containing 50 mM Tris, 500 mM NaCl, and 250 mM imidazole (pH 7.4). Eluted fractions were dialyzed against D-PBS using Slide-A-Lyzer G3 Dialysis Cassettes (10 kDa MWCO; Thermo Fisher Scientific). Protein purity was verified by SDS-PAGE, and concentrations were determined using the Pierce^TM^ Bradford Protein Assay Kit (Thermo Fisher Scientific). 

### 4.5. Fluorescence Microscopy

HeLa cells were seeded at 2 × 10^4^ cells per well in 8-well chamber slides (Millicell EZ Slide, Sigma-Aldrich, St. Louis, MO, USA; or µ-Slide 8 Well glass-bottom, ibidi, Gräfelfing, Germany) and allowed to adhere for 24 h. For assessment of intrinsic cell-penetrating ability, the culture medium was replaced with free TAMRA or TAMRA–Nuc1510 at 5 µM prepared in DMEM supplemented with 10% FBS. For assessment of cargo delivery, cells were treated with AG, AG–Nuc1511, AG–Nuc1510, or AG–TAT at 1, 2, or 3 µM prepared in DMEM with 10% FBS. All cargo delivery treatments were performed at 37 °C, and the highest concentration (3 µM) was additionally tested at 4 °C to assess the contribution of energy-dependent uptake pathways.

TAMRA–Nuc1510 samples were incubated for 1 h, 4 h, or overnight at 37 °C, whereas all AG–CPP samples were incubated overnight only. Following incubation, cells were washed with PBS and mounted with ProLong^TM^ Diamond Antifade Reagent containing DAPI (Invitrogen, Waltham, MA, USA) according to the manufacturer’s instructions. For the 4 °C condition, cells were equilibrated at 4 °C for 30 min prior to treatment, maintained at 4 °C throughout the incubation period, and washed with ice-cold PBS before mounting. Fluorescence imaging was performed using a Keyence BZ-X800 (Keyence, Osaka, Japan) digital fluorescence microscope. For each AG–CPP construct, the green-channel exposure time was optimized for signal detection at the 3 µM condition and kept constant across the remaining concentrations of the same construct. Because the intracellular AG signal varied along the *Z*-axis, focus was set using the nuclear DAPI signal to ensure that images were acquired at a consistent focal plane within the cell. Single-section optical images were acquired using the structured illumination-based sectioning function, and image analysis was performed using BZ-X800 Analyzer software (version 1.1.1.8) and FIJI (version 2.16.0). Fluorescence filter specifications are listed in Table S2.

### 4.6. Flow Cytometry

HeLa cells were seeded at 3 × 10^4^ cells per well in 48-well plates (Nunc^TM^ Cell-Culture Treated Multidishes; Thermo Fisher Scientific) and allowed to adhere overnight. Cells were then treated with 3 µM AG, AG–Nuc1511, AG–Nuc1510, or AG–TAT as described in Section 4.5. Following overnight incubation, cells were washed with PBS, washed twice with acidic glycine–NaCl solution (0.2 M glycine, 0.15 M NaCl, pH 3.0) on ice for 30 s per wash, and washed again with PBS. Cells were detached using 0.05% (w/v) trypsin–0.53 mM EDTA-4Na solution (Fujifilm, Osaka, Japan), and trypsin was neutralized by addition of DMEM supplemented with 10% FBS. Flow cytometry was performed using the On-Chip Sort HS (On-Chip Biotechnologies, Tokyo, Japan), with a minimum of 5000 events recorded per sample. Data were analyzed using FlowJo (version 10.7.1). All conditions were performed in triplicate.

### 4.7. Cell Viability Assay

HeLa cells were seeded at 1 × 10^4^ cells per well in 96-well microplates (Nunc^TM^ MicroWell^TM^; Thermo Fisher Scientific) and allowed to adhere overnight. The culture medium was replaced with AG–Nuc1511 or AG–Nuc1510 at 1, 3, 5, or 10 µM prepared in DMEM supplemented with 10% FBS. Cell viability was assessed by MTT assay. Following 24 h incubation, treatment solutions were removed and cells were washed with PBS. A mixture of 50 µL MTT solution (Fujifilm) (5 mg/mL in PBS) and 50 µL Opti-MEM I Reduced Serum Medium (Thermo Fisher Scientific) was added to each well, and plates were incubated at 37 °C for 3 h. The medium was then removed, and formazan crystals were dissolved by addition of 150 µL MTT solvent (4 mM HCl, 0.1% NP-40 substitute in isopropanol), followed by 30 min agitation on an orbital shaker protected from light, with additional pipetting to ensure complete dissolution. Absorbance was measured at 590 nm. All conditions were performed in triplicate.

### 4.8. Statistical Analysis

Statistical significance was assessed using Welch’s *t*-test to compare flow cytometry fluorescence intensities and MTT cell viability values across CPP treatment concentrations relative to untreated controls.

## 5. Conclusions

In this study, we demonstrated that two novel chimeric CPPs, Nuc1511 and Nuc1510, effectively facilitate intracellular delivery of Azami Green protein into HeLa cells via covalent conjugation, achieving >80-fold and ~9-fold increases in intracellular fluorescence relative to unconjugated AG, respectively, and substantially outperforming the well-established TAT peptide. Both peptides demonstrated favorable cytotoxicity profiles at delivery-relevant concentrations. These results establish Nuc1511 and Nuc1510 as promising candidates for intracellular protein delivery, with Nuc1511 showing particularly high efficiency, warranting further investigation into their mechanistic basis and broader cargo applicability.

## Figures and Tables

**Figure 1:**
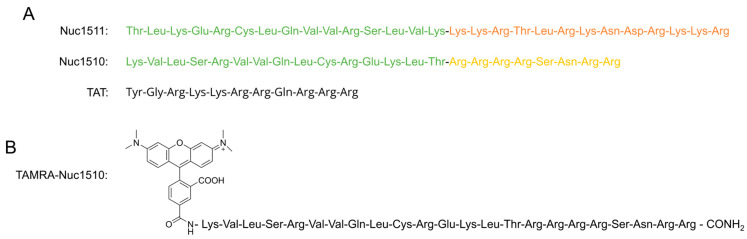
Sequences and structure of the CPPs used in this study. (**A**) Amino acid sequences of Nuc1511, Nuc1510, and TAT, with color coding indicating different motifs: green, BC-box motif of the VHL protein; orange, LIMK2 nucleolar localization signal; yellow, a proprietary CPP sequence. (**B**) Schematic structure of TAMRA-labeled Nuc1510.

**Figure 2:**
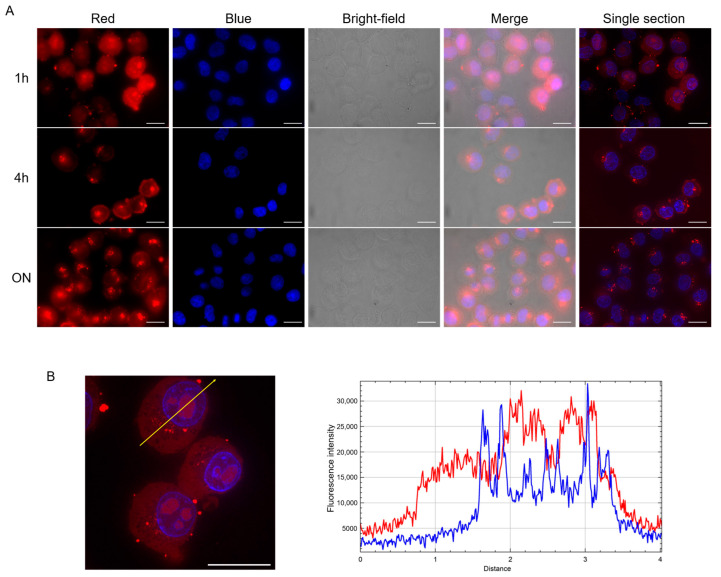
Cellular uptake of TAMRA–Nuc1510 by HeLa cells. (**A**) Fluorescence microscopy images of HeLa cells following incubation with 5 µM TAMRA–Nuc1510 for 1 h, 4 h, or overnight. Free TAMRA served as the negative control (Figure S1). Scale bar: 20 µm. (**B**) Single-section image of a HeLa cell incubated with TAMRA–Nuc1510 for 1 h with a line scan (**left**); the yellow arrow indicates the line scan. Scale bar: 20 µm. Fluorescence intensity profiles of TAMRA and DAPI along the scan line (**right**). Red: TAMRA, blue: DAPI.

**Figure 3:**
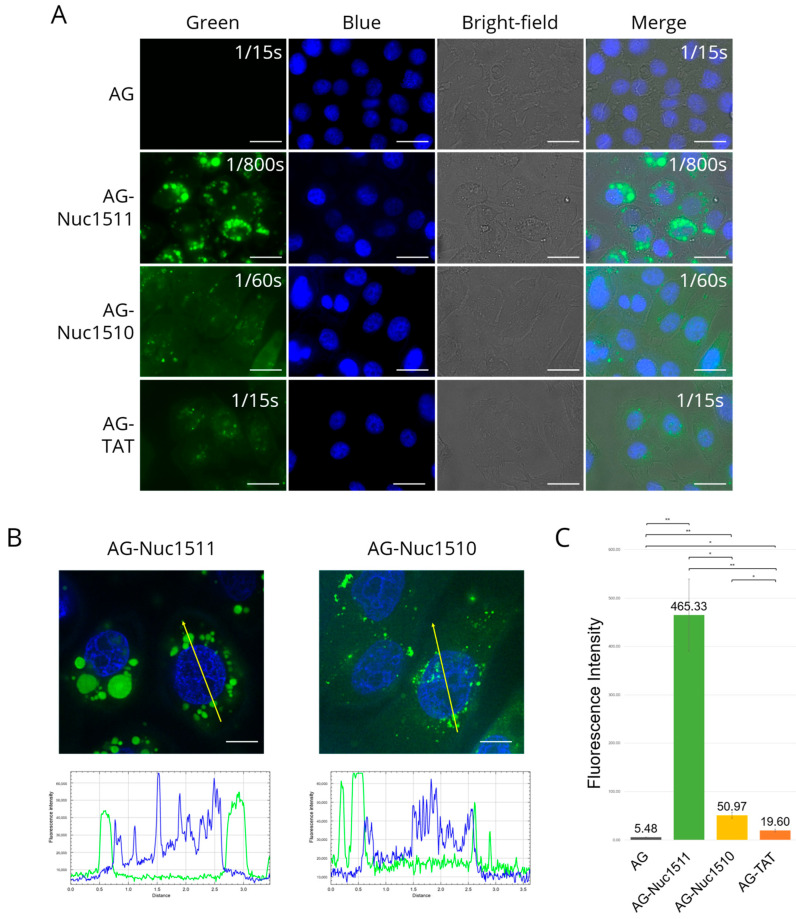
Cellular delivery of AG–CPP fusion proteins in HeLa cells. (**A**) Fluorescence microscopy images of HeLa cells following overnight incubation with 3 µM AG, AG–Nuc1511, AG–Nuc1510, or AG–TAT at 37 °C. Exposure time for the green channel is indicated in the top right of the relevant images. Scale bar: 20 µm. (**B**) Single-section images of HeLa cells treated with 3 µM AG–Nuc1511 or AG–Nuc1510; the yellow arrow indicates the line scan. Scale bar: 20 µm. Bottom panels show the corresponding fluorescence intensity profiles along the scan line. Green: Azami Green, blue: DAPI. (**C**) Quantification of intracellular AG fluorescence intensity by flow cytometry following overnight incubation with 3 µM AG, AG–Nuc1511, AG–Nuc1510, or AG–TAT (*n* = 3). Statistical significance was assessed by Welch’s *t*-test; *: *p* < 0.05, **: *p* < 0.01.

**Figure 4:**
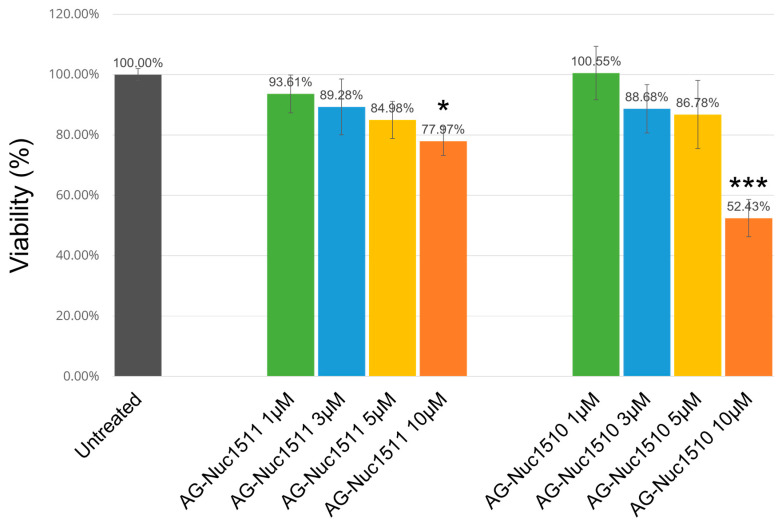
Cytotoxicity of AG–Nuc1511 and AG–Nuc1510 in HeLa cells evaluated by MTT assay, expressed as a percentage relative to untreated cells (100%), (*n* = 3, error bars represent standard deviation). Statistical comparisons were made against untreated controls using Welch’s *t*-test; *: *p* < 0.05, ***: *p* < 0.001.

## Data Availability

The data produced in this study are available from the corresponding author upon reasonable request.

## Supplementary Materials

The following supporting information can be downloaded at: https://www.mdpi.com/article/10.3390/ijms27156726/s1.

## Abbreviations

The following abbreviations are used in this manuscript:

CPP	Cell-penetrating peptide
TAT	Trans-activator of Transcription
NoLS	Nucleolar localization signal
Nuc	Nucleolaron
FITC	Fluorescein isothiocyanate
TAMRA	5(6)-carboxytetramethylrhodamine
AG	Azami Green
DAPI	4′,6-diamidino-2-phenylindole
SDS-PAGE	Sodium dodecyl sulfate polyacrylamide gel electrophoresis
FBS	Fetal bovine serum
RP-HPLC	Reverse-phase high-performance liquid chromatography
PCR	Polymerase chain reaction
MTT	3-(4,5-dimethylthiazol-2-yl)-2,5-diphenyltetrazolium bromide
EGFP	Enhanced green fluorescent protein
