# Supplementary material for: Nucleolaron1511 and Nucleolaron1510 Facilitate Effective Intracellular Delivery of Azami Green Protein in HeLa Cells
Source: Int J Mol Sci. 2026 Jul 28;27(15):6726. doi: 10.3390/ijms27156726 (PMC13466294; doi:10.3390/ijms27156726)
Supplement: Supplementary file 1 [file ijms-27-06726-s001.zip › ijms-4374405-supplementary.pdf]

(Supplementary materials)

Nucleolaron1511 and Nucleolaron1510 Facilitate Effective Intracellular Delivery of Azami Green Protein in HeLa Cells

Khai Nguyen[1, 2, 3], Ryo Yonezawa[1, 3], Nahoko Bailey-Kobayashi[4], Tetsuhiko Yoshida[4], Shuichi Asakawa[1, 3*]

[1] Department of Aquatic Bioscience, Laboratory of Aquatic Molecular Biology and Biotechnology, Graduate School of Agricultural and Life Sciences, The University of Tokyo, Bunkyo, Tokyo, Japan.
[2] International Program in Agricultural Development Studies, Graduate School of Agricultural and Life Sciences, The University of Tokyo, Bunkyo, Tokyo, Japan.
[3] Department of Aquatic Bioscience, Signal Peptidome Research Laboratory, Graduate School of Agricultural and Life Sciences, The University of Tokyo, Bunkyo, Tokyo, Japan.
[4] Institute for Advanced Sciences, Toagosei Co., Ltd., Tsukuba, Japan
* Correspondence: asakawa@g.ecc.u-tokyo.ac.jp

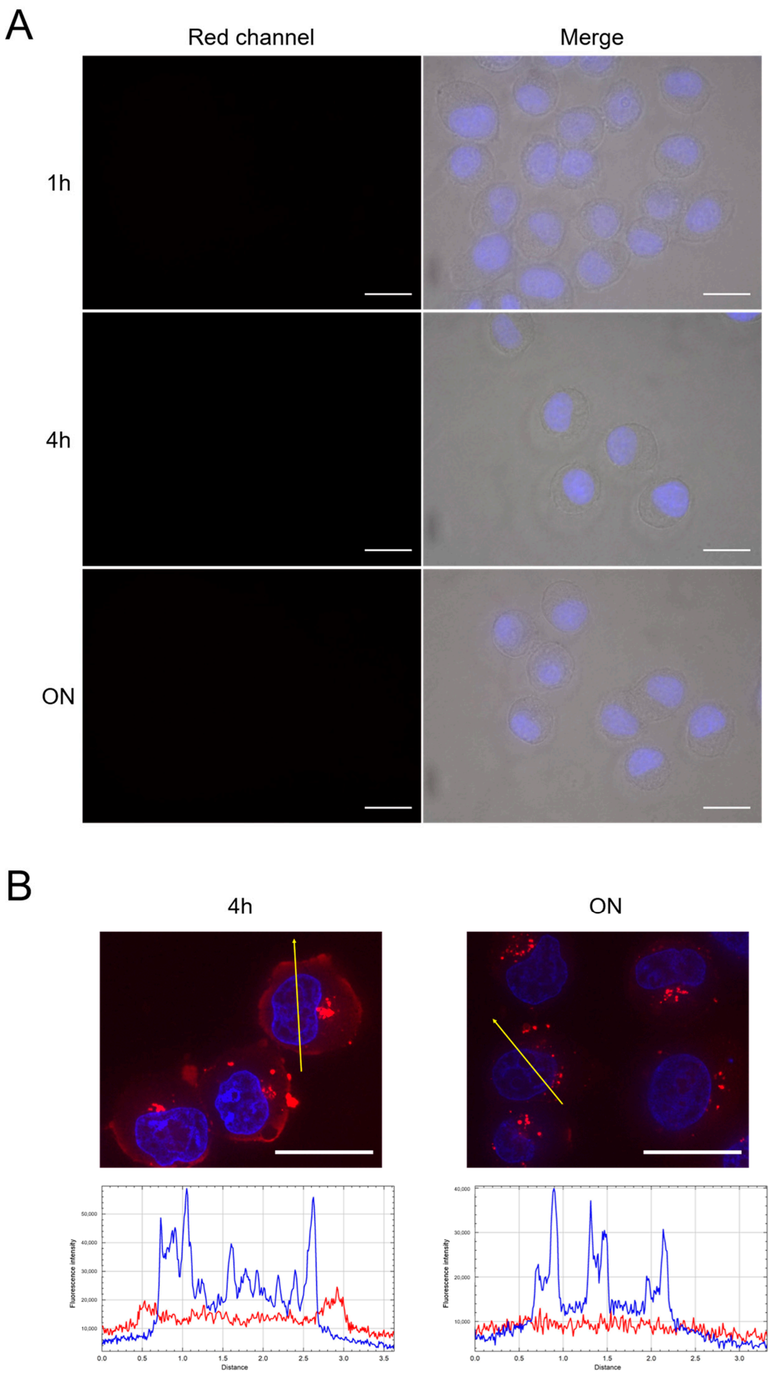

Figure S1. Free TAMRA and TAMRA–Nuc1510 uptake images. (A) HeLa cells incubated with 5 µM of free TAMRA for 1 h, 4 h, or overnight. Scale bar: 20 µm. (B) Single-section images of HeLa cells incubated with 5 µM of TAMRA-labeled Nuc1510 for 4 h (left) or overnight (right), with the corresponding fluorescence intensity profiles. Red lines indicate TAMRA signal, and blue lines indicate DAPI signal. Scale bar: 20 µm.

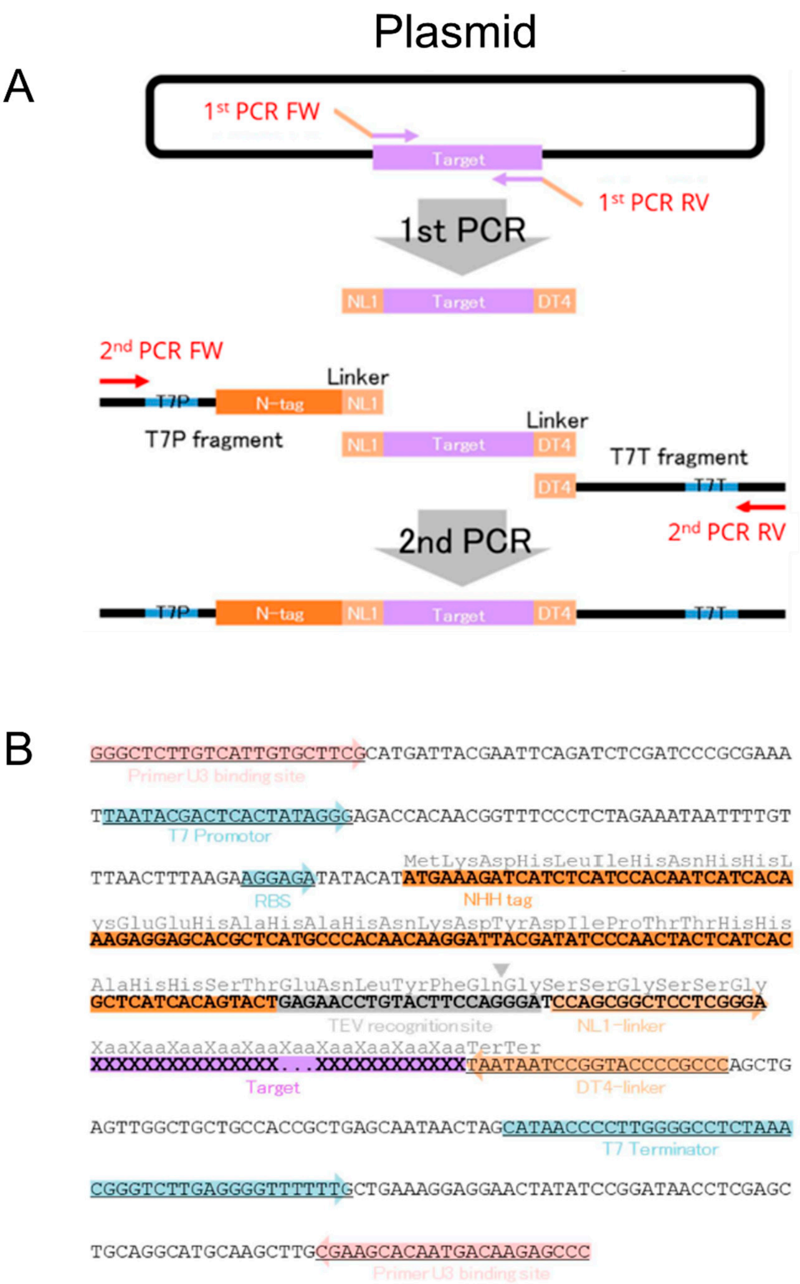

Figure S2. Two-step PCR strategy for DNA cassette preparation. (A) Schematic representation of the two-step PCR process used to generate DNA cassettes for cell-free in vitro translation, adapted with permission from Yabuki et al. [38]; (B) annotated sequence of the two-step PCR product, indicating the U3 primer binding sites, T7 promoter and T7 terminator regulatory elements, ribosome binding site (RBS), NHH purification tag for anti-His magnetic bead capture, and TEV protease recognition site for optional fusion protein cleavage (cleavage was not performed in this study).

Table S1. Primer sequences

**Primer**	**Sequence**
First PCR Fw (Common for AG, AG-Nuc1511, AG-Nuc1510)	GATCCAGCGGCTCCTCGGGAATGTCGGTAATCAAG
1st PCR RV (AG)	GGTACCGGATTATTAACTCCCACCACCCCCAGTT
1st PCR RV (AG-Nuc1511)	CGGGGTACCGGATTATTAGCGTTTTTTGCGATCGTT
1st PCR RV (AG-Nuc1510)	CGGGGTACCGGATTATTAACGGCGATTGCTGCGAC
2nd PCR FW/RV (Primer U3) (AG, AG-Nuc1511, AG-Nuc1510, AG-TAT)	GGGCTCTTGTCATTGTGCTTCG

Complete amino acid sequences of AG-CPP constructs

Blue: N-terminal T7P; green: monomeric Azami Green sequence; black: linker sequence; orange: cell penetrating peptide sequence

1. AG

MKDHLIHNHHKEEHAHAHNKDYDIPTTHHAHHSTENLYFQGSSGSSGMSVIKPEMKIKLCMRGTVNGHNFVIE
GEGKGNPYEGTQILDLNVTEGAPLPFAYDILTTVFQYGNRAFTKYPADIQDYFKQTFPEGYHWERSMTYEDQG
ICTATSNISMRGDCFFYDIRFDGTNFPPNGPVMQKKTLKWEPSTEKMYVRDGVLKGDVNMALLLEGGGHYRCD
FKTTYKAKKDVRLPDAHKVDHRIEILKHDKDYNKVKLYENAVARYSMLPSQAKGLQQGGTGGGGS

2. AG-Nuc1511

MKDHLIHNHHKEEHAHAHNKDYDIPTTHHAHHSTENLYFQGSSGSSGMSVIKPEMKIKLCMRGTVNGHNFVIE
GEGKGNPYEGTQILDLNVTEGAPLPFAYDILTTVFQYGNRAFTKYPADIQDYFKQTFPEGYHWERSMTYEDQG
ICTATSNISMRGDCFFYDIRFDGTNFPPNGPVMQKKTLKWEPSTEKMYVRDGVLKGDVNMALLLEGGGHYRCD
FKTTYKAKKDVRLPDAHKVDHRIEILKHDKDYNKVKLYENAVARYSMLPSQAKGLQQGGTGGGGSTLKERCLQ
VVRSLVKKKRTLRKNDRKKR

3. AG-Nuc1510

MKDHLIHNHHKEEHAHAHNKDYDIPTTHHAHHSTENLYFQGSSGSSGMSVIKPEMKIKLCMRGTVNGHNFVIE
GEGKGNPYEGTQILDLNVTEGAPLPFAYDILTTVFQYGNRAFTKYPADIQDYFKQTFPEGYHWERSMTYEDQG
ICTATSNISMRGDCFFYDIRFDGTNFPPNGPVMQKKTLKWEPSTEKMYVRDGVLKGDVNMALLLEGGGHYRCD
FKTTYKAKKDVRLPDAHKVDHRIEILKHDKDYNKVKLYENAVARYSMLPSQAKGLQQGGTGGGGSKVLSRVVQ
LCREKLTRRRRSNRR

4. AG-TAT

MKDHLIHNHHKEEHAHAHNKDYDIPTTHHAHHSTENLYFQGSSGSSGMSVIKPEMKIKLCMRGTVNGHNFVIE
GEGKGNPYEGTQILDLNVTEGAPLPFAYDILTTVFQYGNRAFTKYPADIQDYFKQTFPEGYHWERSMTYEDQG
ICTATSNISMRGDCFFYDIRFDGTNFPPNGPVMQKKTLKWEPSTEKMYVRDGVLKGDVNMALLLEGGGHYRCD
FKTTYKAKKDVRLPDAHKVDHRIEILKHDKDYNKVKLYENAVARYSMLPSQAKGLQQGGTGGGGSYGRKKRRQ
RRR

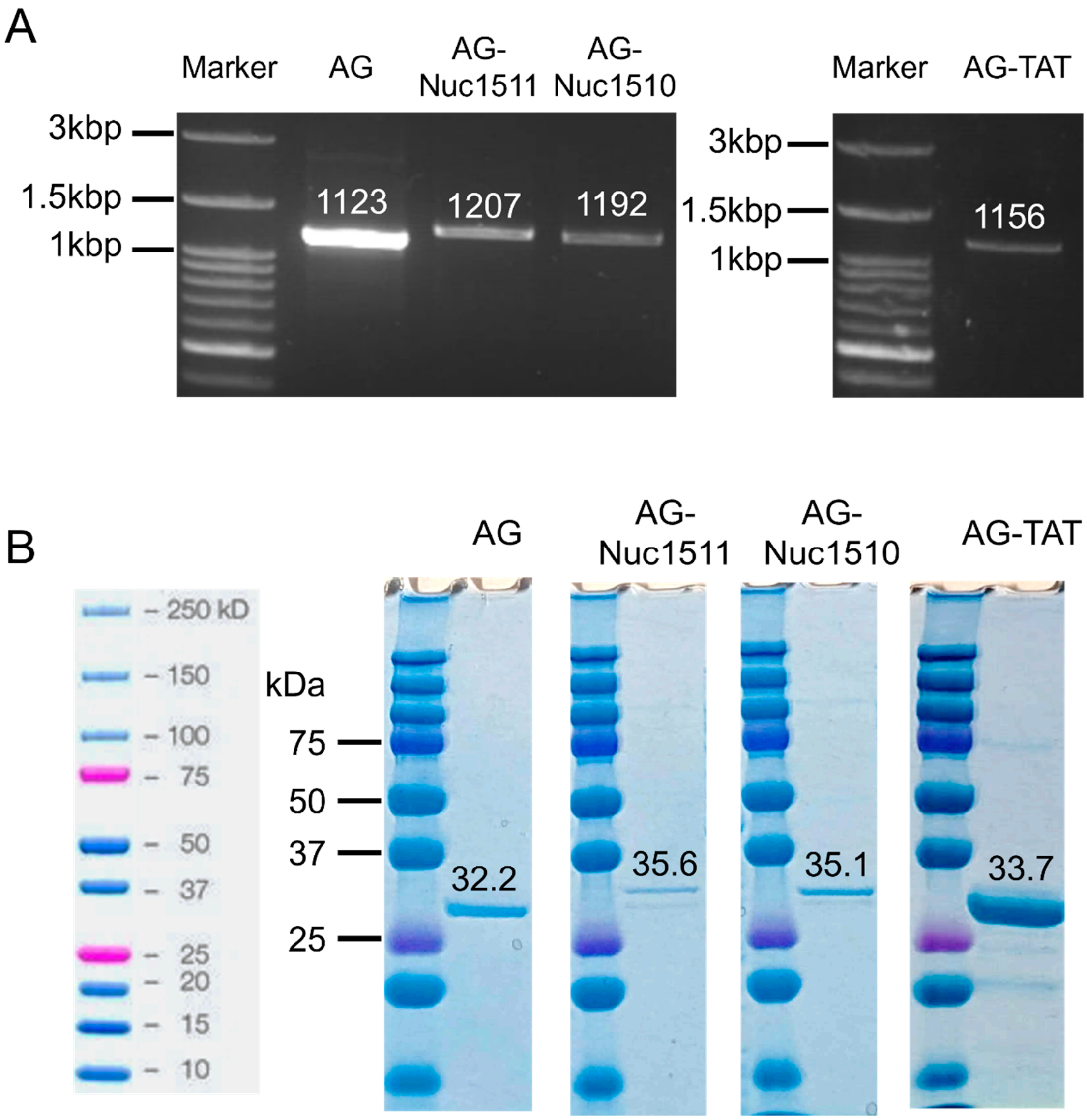

Figure S3. Production and purification of AG–CPP fusion proteins. (A) Agarose gel electrophoresis of DNA cassettes confirming successful assembly of the AG, AG–Nuc1511, AG–Nuc1510, and AG–TAT templates. Numbers above each band indicate the size of the final construct inclusive of the target protein sequence and T7 promoter and terminator fragments; (B) SDS-PAGE of purified recombinant proteins confirming successful cell-free translation and purification of all four constructs. Numbers above each band indicate the molecular weight of the corresponding fusion protein construct.

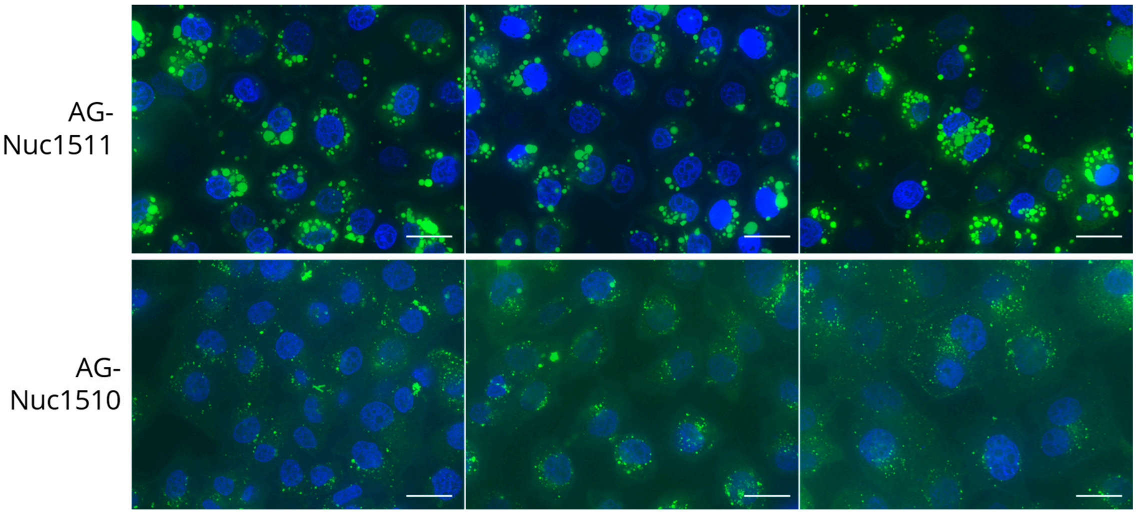

Figure S4. Single-section images of HeLa cells treated with 3 μM AG–Nuc1511 or AG–Nuc1510. Scale bar: 20 μm.

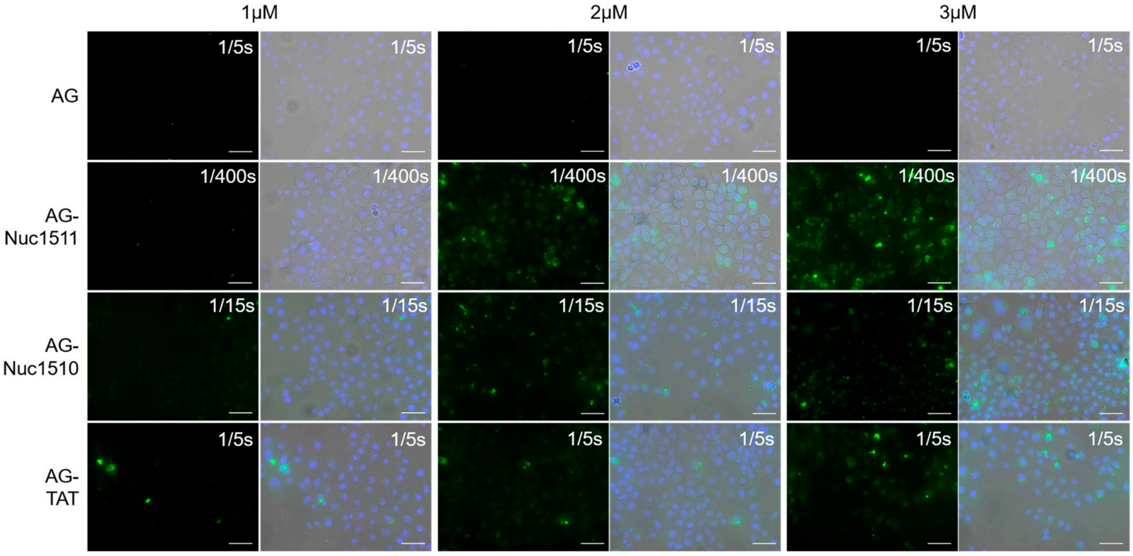

Figure S5. Fluorescence microscopy images of HeLa cells incubated with 1, 2, or 3 μM of AG or AG-CPP constructs. Exposure time for the green channel is indicated in the top right of each image. Scale bar: 50 μm.

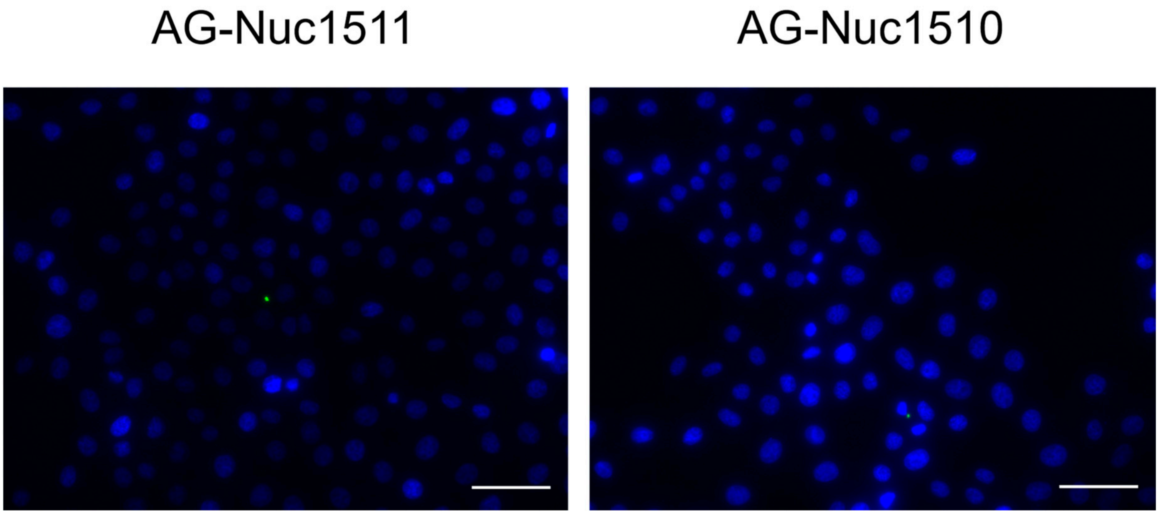

Figure S6. Fluorescence microscopy images of HeLa cells following overnight incubation with 3 μM of AG–Nuc1511 or AG–Nuc1510 at 4°C. Exposure time for the green channel was 1/12s for both images. Scale bar: 50 μm.

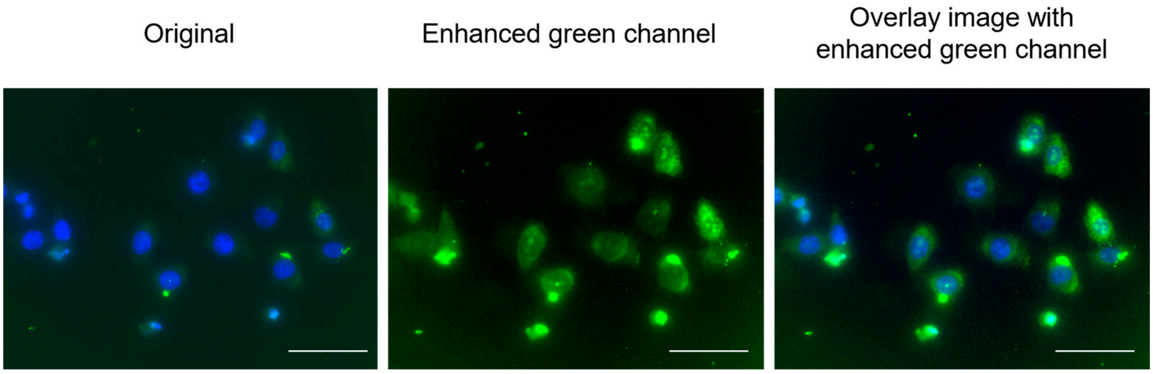

Figure S7. Subcellular localization of AG–TAT in HeLa cells treated with 3 μM AG–TAT. Middle and right panels show brightness-enhanced green channel images. Scale bar: 50 μm.

Table S2. Keyence BZ-X800 microscope's fluorescence filters

**Filter name**	**Target signal**	**Excitation wavelength**	**Detected emission wavelength**	**Dichroic mirror wavelength**
BZ-X filter DAPI	DAPI	360/40	460/50	400
BZ-X filter GFP	Azami Green	470/40	525/50	495
BZ-X filter TexasRed	TAMRA	560/40	630/75	585
